# Potential pre-industrial–like new particle formation induced by pure biogenic organic vapors in Finnish peatland

**DOI:** 10.1126/sciadv.adm9191

**Published:** 2024-04-03

**Authors:** Wei Huang, Heikki Junninen, Olga Garmash, Katrianne Lehtipalo, Dominik Stolzenburg, Janne L. P. Lampilahti, Ekaterina Ezhova, Simon Schallhart, Pekka Rantala, Diego Aliaga, Lauri Ahonen, Juha Sulo, Lauriane L. J. Quéléver, Runlong Cai, Pavel Alekseychik, Stephany B. Mazon, Lei Yao, Sara M. Blichner, Qiaozhi Zha, Ivan Mammarella, Jasper Kirkby, Veli-Matti Kerminen, Douglas R. Worsnop, Markku Kulmala, Federico Bianchi

**Affiliations:** ^1^ Institute for Atmospheric and Earth System Research / Physics, Faculty of Science, University of Helsinki, Helsinki 00014, Finland.; ^2^ Institute of Physics, University of Tartu, Tartu 50411, Estonia.; ^3^ Aerosol Physics Laboratory, Physics Unit, Tampere University, Tampere 33720, Finland.; ^4^ Atmospheric Composition Unit, Finnish Meteorological Institute, Helsinki 00101, Finland.; ^5^ Institute for Materials Chemistry, TU Wien, 1060 Vienna, Austria.; ^6^ Bioeconomy and Environment, Natural Resources Institute Finland, Helsinki 00790, Finland.; ^7^ Shanghai Key Laboratory of Atmospheric Particle Pollution and Prevention (LAP3), Department of Environmental Science & Engineering, Fudan University, Shanghai 200438, China.; ^8^ Department of Environmental Science, Stockholm University, Stockholm 11418, Sweden.; ^9^ Bolin Centre for Climate Research, Stockholm University, Stockholm 11418, Sweden.; ^10^ Joint International Research Laboratory of Atmospheric and Earth System Research, School of Atmospheric Sciences, Nanjing University, Nanjing 210023, China.; ^11^ Institute for Atmospheric and Environmental Sciences, Goethe University Frankfurt, Frankfurt am Main 60438, Germany.; ^12^ CERN, the European Organization for Nuclear Research, CH-1211 Geneve 23, Switzerland.; ^13^ Aerodyne Research Inc., Billerica, MA 01821, USA.

## Abstract

Pure biogenic new particle formation (NPF) induced by highly oxygenated organic molecules (HOMs) could be an important mechanism for pre-industrial aerosol formation. However, it has not been unambiguously confirmed in the ambient due to the scarcity of truly pristine continental locations in the present-day atmosphere or the lack of chemical characterization of NPF precursors. Here, we report ambient observations of pure biogenic HOM-driven NPF over a peatland in southern Finland. Meteorological decoupling processes formed an “air pocket” (i.e., a very shallow surface layer) at night and favored NPF initiated entirely by biogenic HOM from this peatland, whose atmospheric environment closely resembles that of the pre-industrial era. Our study sheds light on pre-industrial aerosol formation, which represents the baseline for estimating the impact of present and future aerosol on climate, as well as on future NPF, the features of which may revert toward pre-industrial–like conditions due to air pollution mitigation.

## Teaser

Nighttime pure biogenic new particle formation was observed in an environment closely resembling the pre-industrial atmosphere.

## INTRODUCTION

More than half of cloud condensation nuclei (CCN) are thought to originate from new particle formation (NPF) processes (*1*), which can be initiated by clustering of sulfuric acid and stabilizing bases such as ammonia, amines, highly oxygenated organic molecules (HOMs), or by iodine oxoacids (*2*–*4*), with sulfuric acid most often believed to be an essential component for the early stages of NPF processes (*5*). Recent studies from the CLOUD (Cosmics Leaving OUtdoor Droplets) chamber facility at CERN (European Organization for Nuclear Research) have shown that HOM—formed through fast auto-oxidation reactions of volatile organic compounds (VOCs; e.g., monoterpenes) (*6*)—can drive nucleation without sulfuric acid at atmospheric conditions (*7*). This could be an important nucleation mechanism in pristine environments unaffected by anthropogenic pollution. However, in the present anthropogenically perturbed atmosphere, it is extremely difficult to find truly pristine environments on the continents to study the importance of pure biogenic HOM-driven mechanism for pre-industrial aerosol formation (*8*).

Most of the “remote” sites reported previously [e.g., Nieminen *et al.* (*9*)] are in regions with substantial anthropogenic perturbation (*10*). Even for remote mountain sites, such as the high-altitude research station Jungfraujoch (*11*), the Himalaya (*12*), and the Bolivian Andes (*13*), where NPF events were suspected to be induced only by organics, either the source of HOM precursors cannot be confirmed as entirely biogenic (*11*); or HOM dimers or larger molecules (crucial for pure organic nucleation) are absent (*12*); or sulfuric acid remains an important driver of NPF in contrast to previous expectations (*13*). A recent study in the boreal forest in Hyytiälä, Finland showed that charged HOM from monoterpene ozonolysis induced nighttime ion cluster formation, although new particles did not grow beyond a few nanometers (*14*). Also, participation of sulfuric acid in the NPF could not be completely ruled out due to higher sulfuric acid concentrations than those reported in the pure biogenic nucleation experiments at CLOUD (*7*, *14*). Terpene emissions in the boreal wetlands in Siikaneva, Finland were found to initiate stronger atmospheric nighttime NPF than was typically observed over the nearby Hyytiälä boreal forests, contributing to a cooling effect compensating the warming effect from wetland methane emissions, which should be considered when estimating the wetland aerosol formation impact in the future climate. However, the actual nucleation mechanism and the chemistry behind the nighttime NPF were not investigated or discussed, although the minor role of nitrogen-containing HOM and sulfuric acid in the particle formation and growth process was concluded (*15*). NPF events, frequently observed at nighttime and daytime in the subboreal forest of North America, were speculated to be initiated by HOM formed from monoterpenes due to the absence of identifiable sources of sulfuric acid precursor species. But this cannot be confirmed without gas-phase NPF precursor measurements at that measurement site (*10*). In addition, for NPF events, which were predominantly driven by organics, NPF precursor chemistry might still be anthropogenically perturbed, e.g., via NO*_x_*, because NO*_x_* or NO_3_ radicals [derived from NO*_x_* (*16*)] suppress the α-pinene HOM-induced NPF and early particle growth by producing HOM with higher volatilities, through inhibiting formation of dimers (*17*) or ultra-low volatility organic compounds (*18*) (ULVOCs; see details in Materials and Methods). This might also be the case for nighttime clustering and NPF events reported in the other locations, where NPF precursor measurements were mostly unavailable (*14*, *19*, *20*). Thus, despite remarkable progress in understanding the role of HOM in NPF in laboratory and field studies, there are no unambiguously confirmed ambient observations of pure biogenic HOM induced NPF in conditions representative of the pre-industrial era with minimal anthropogenic pollution.

## RESULTS

**Fig. 1.**
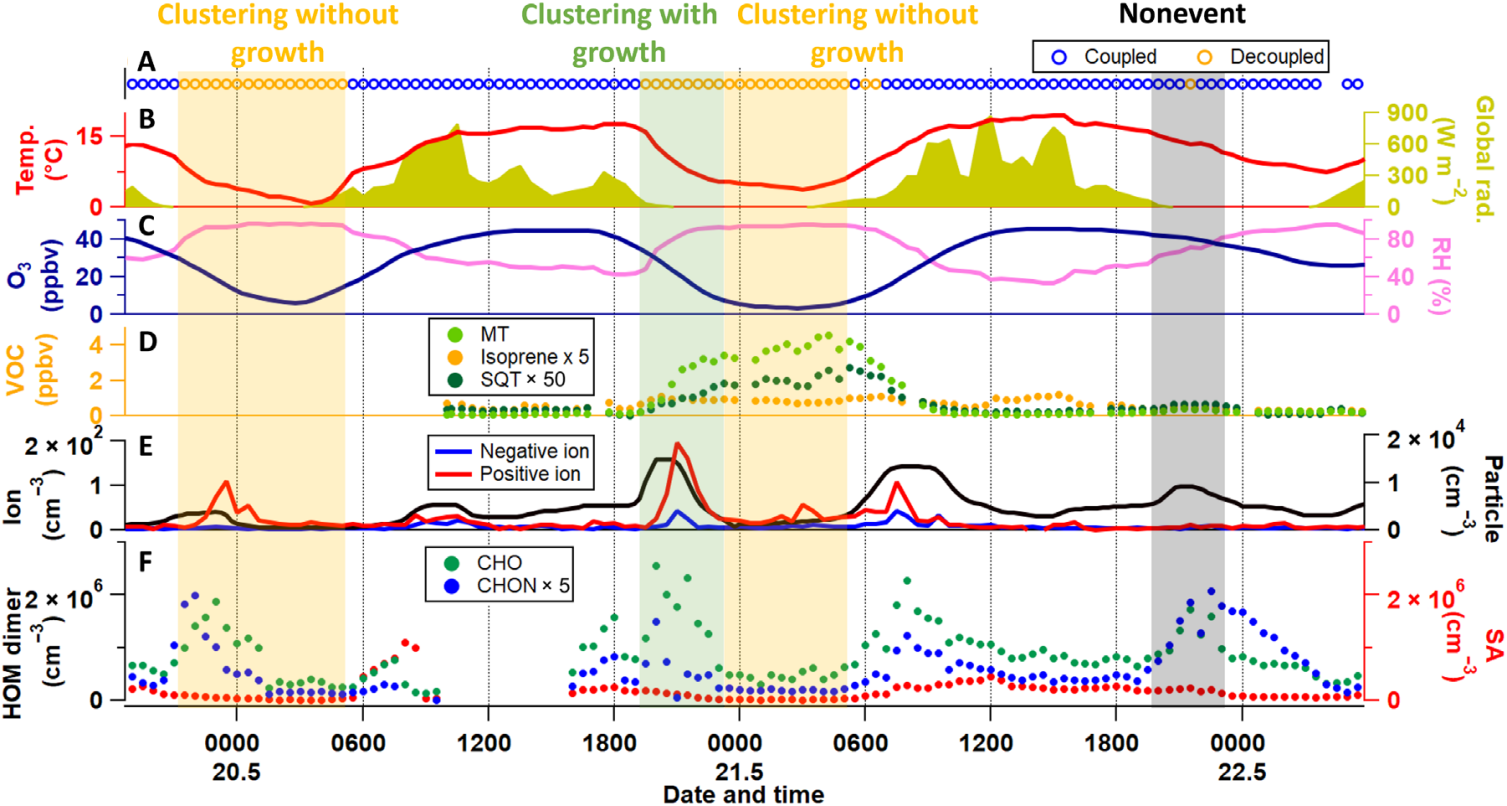
Three noctural situations representative of Siikaneva. Data are from 19 to 22 May 2016, during which we observed a clustering with growth event (20 May 1900 to 2300), two clustering without growth events (19 May 2200 to 0500 and 20 May 2300–0500), and a nonevent (21 May 2000 to 2300). (**A**) Surface layer state. (**B**) Temperature and global radiation. (**C**) Mixing ratios of O_3_ and relative humidity (RH). (**D**) mixing ratios of isoprene, monoterpenes (MTs), and sesquiterpenes (SQTs) measured with a proton transfer reaction time-of-flight (PTR-TOF) mass spectrometer. (**E**) Number concentrations of intermediate ions (2 to 4 nm) and atmospheric clusters (1.1 to 2.5 nm), measured with a neutral cluster and air ion spectrometer (NAIS) and a particle size magnifier (PSM), respectively. (**F**) HOM dimer and sulfuric acid (SA) concentration measured with the CI-APi-TOF. These neutral gas-phase precursor vapors could form clusters and grow into nanoparticles after stabilization and activation (*5*). All data are reported in eastern European time (UTC +2).

Here, we report ambient particle formation from pure biogenic organic vapors in the nocturnal continental surface layer over a peatland area. The data were part of the campaign performed between 10 March and 20 June 2016, at Siikaneva peatland in southern Finland. Siikaneva peatland has been found to be a large source of terpenes (dominated by isoprene, α-pinene, etc.) with more than 90% of the flux within the peatland area and negligible contribution from the surrounding Scots pine forests (*21*). These peatland terpene emissions initiate stronger atmospheric NPF in Siikaneva than is typically observed over boreal forests, contributing to the regional aerosol number budget (*15*). In addition, a stratified boundary layer occurs in Siikaneva during nights characterized by clear skies and low wind speeds (*15*) (40% of the nights; see also fig. S1A). The strong inversions on these nights hinder turbulent mixing, causing the lowest surface layer above the peatland (up to few meters high) to be decoupled from the rest of the nocturnal boundary layer (*15*). These inversions were monitored by the turbulence level and CO_2_ and CH_4_ concentrations at a 3-m height (see details in Materials and Methods) (*15*). Initial gases (e.g., O_3_) or anthropogenic pollutants (e.g., NO*_x_*) were largely depleted by dry deposition to the wet peatland surface inside the shallow decoupled layer (i.e., “air pocket”) (*22*–*24*). In contrast, peatland emissions such as terpenes were trapped and concentrated within the layer (see Fig. 1, C and D, and fig. S2; and also a decoupling example in the nearby (~5 km away) boreal forest in Hyytiälä in fig. S3) (*15*). The sharp decrease of O_3_ mixing ratios [partially also due to chemical loss (*22*, *24*)] and increase of terpene concentrations suggest oxidation processes and resulting formation of oxygenated organic molecules including HOM.

### Nighttime NPF events in Siikaneva

During the measurement period, nighttime NPF events were frequently observed, starting after sunset and lasting for a few hours, coincident with meteorological decoupling due to strong near-surface cooling (Fig. 1, A and B, and fig. S2). To chemically and physically characterize the early stages of these NPF events, we used a nitrate chemical ionization atmospheric pressure interface time-of-flight (CI-APi-TOF) mass spectrometer to measure neutral gas-phase precursor vapors (e.g., sulfuric acid and HOM), as well as particle counters to detect number concentrations and size distributions of ambient ions, clusters, and particles (see Materials and Methods for instrumental details). These neutral gas-phase precursor vapors could form clusters and grow into nanoparticles after stabilization and activation (*5*). The formed particles were found to grow further, at least to several tens of nanometers (reaching at least to the instrument upper detection limit of 42 nm, which could be relevant to at least the local climate), during some of the events (nine events in total identified in May; see fig. S4 and table S1; hereafter called clustering with growth events). These events were characterized by a clear increase of intermediate ions (2 to 4 nm) and atmospheric clusters (1.1 to 2.5 nm; see Fig. 1E and figs. S2 and S5), as well as non-nitrated HOM dimers (CHO HOM dimers, C_17–20_H_26–32_O_7–18_; see Fig. 1F and fig. S2) that likely originate from the ozonolysis of monoterpenes. However, during some other event nights, particles did not grow above ~10 nm after the clustering process (see table S1; hereafter called clustering without growth events). These nights with limited growth showed either no notable increase of the CHO HOM dimers (see, e.g., 21 May 0000 to 0500 and 20 May 0100 to 0500 in Fig. 1F) or a substantial increase of nitrated HOM dimers (CHON HOM dimers, C_19–20_H_29–32_O_9–18_ N_1–2_; see, e.g., 19 May 2200 to 0100 in Fig. 1F), which are possibly formed from NO_3_ radical oxidation of monoterpenes (*25*). This type of nocturnal events were also observed in the nearby forest in Hyytiälä (*14*, *26*), which were found to be connected to HOM produced by monoterpene ozonolysis (*14*, *26*). On nights when no cluster formation was observed at all (see table S1; hereafter called nonevents), the surface layer was in a coupled condition (Fig. 1A and fig. S2) and there was a clear increase of CHON HOM dimers (see, e.g., 21 May 2000 to 2300 in Fig. 1F) (*15*). Note that the small increase in the concentrations of atmospheric clusters during the nonevent nights (Fig. 1E and fig. S2) has been shown to be associated with HOM dimers, for clusters down to, e.g., 1.1 nm in size, particularly (*27*). The sulfuric acid level was found to be always lower than the CHO HOM dimer concentration during decoupled nights, indicating negligible sulfuric acid production at decoupled nights from photochemical (*28*) or Criegee intermediate reactions (*29*).

### Composition of NPF precursors

**Fig. 2.**
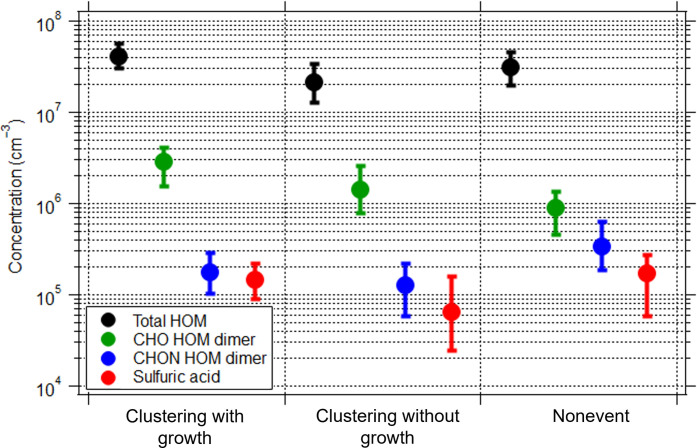
Concentration of neutral precursor species in the three types of nocturnal situations observed in Siikaneva. Shown are median concentrations of total HOM (mass-to-charge > 300 Th), non-nitrated HOM dimers (CHO HOM dimers), nitrated HOM dimers (CHON HOM dimers), and sulfuric acid for decoupled clustering with growth events, decoupled clustering without growth events, and coupled nonevents. Lower and upper error bars represent 25th and 75th percentiles, respectively.

Figure 2 compares the concentrations of total HOM, CHO HOM dimers, CHON HOM dimers, and sulfuric acid for the above-mentioned three types of nocturnal situations (i.e., clustering with growth, clustering without growth, and nonevents). During both types of clustering events, we observed very low median concentrations of sulfuric acid (below 1.5 × 10^5^ cm^−3^; Fig. 2) and also low sulfuric acid ion signals in the ion composition measured by an APi-TOF mass spectrometer (fig. S6C). In addition, large charged sulfuric acid clusters, such as sulfuric acid tetramer that is detected only in the presence of ammonia (*3*), or sulfuric acid clusters with ammonia and dimethylamine (*2*) were also absent (fig. S6A). Sulfuric acid concentrations during the clustering events in Siikaneva (<1.5 × 10^5^ cm^−3^) were also much lower than those reported for the similar nocturnal events in the nearby forest in Hyytiälä (a median value of 8.4 × 10^5^ cm^−3^) (*14*). During the nonevent nights, sulfuric acid concentrations were slightly higher (a median value of 1.7 × 10^5^ cm^−3^) than on nights with clustering, and its ion signals were also higher (fig. S6C). These results indicate that the sulfuric acid–related nucleation process does not play a role alone or synergistically with other precursors in the Siikaneva nighttime events, unlike the NPF mechanism reported in the nearby Hyytiälä forest (*3*, *14*).

In contrast, CHO HOM dimers exhibited the highest median concentration (2.8 × 10^6^ cm^−3^) during clustering with growth events, which are more relevant to at least the local climate (Fig. 2), whereas it was a factor of ~2 lower for clustering without growth events (1.4 × 10^6^ cm^−3^) and lowest for nonevents (8.9 × 10^5^ cm^−3^). Higher levels of total HOM and both negatively and positively charged HOM (*12*) were also seen for clustering with growth events compared to those for clustering without growth events (Fig. 2 and figs. S6B and S7). These results suggest that higher concentrations of low volatility vapors (i.e., HOM for our case) are required to facilitate the further growth of clusters (see also fig. S8). Higher total HOM and CHO HOM dimer concentrations during clustering with growth events are due to higher α-pinene [the dominating monoterpene in Siikaneva (*21*)] reactivity with O_3_ (see fig. S9A) and lower condensation sink (CS) levels (reducing HOM loss from condensation onto background particles; see fig. S9C). This resulted in higher (new) particle number concentrations (Fig. 1E and figs. S2, S4, and S9B). In addition to the difference in the concentrations of CHO HOM dimers (from monoterpenes) between the clustering events with and without growth, other large HOM molecules, such as HOM dimers from other biogenic VOC such as sesquiterpenes (see fig. S2), might also contribute. In comparison, the median concentration of nitrated HOM dimers (i.e., CHON HOM dimers) doubled for nonevents (3.4 × 10^5^ cm^−3^; Fig. 2) compared to both types of clustering events (<1.8 × 10^5^ cm^−3^), as they were subjected to more anthropogenic influences (e.g., NO*_x_*) from outside the peatland (*15*), also consistent with the larger range of CS values (fig. S9c). This led to higher total HOM concentrations for nonevents (a median value of 3.1 × 10^7^ cm^−3^) compared to the clustering without growth events (a median value of 2.1 × 10^7^ cm^−3^; Fig. 2). These results highlight the importance of HOM dimers from monoterpene ozonolysis (i.e., CHO HOM dimers) as opposed to the total HOM in the early growth of small clusters, consistent with laboratory observations (*3*).

### Particle formation and growth rates

**Fig. 3.**
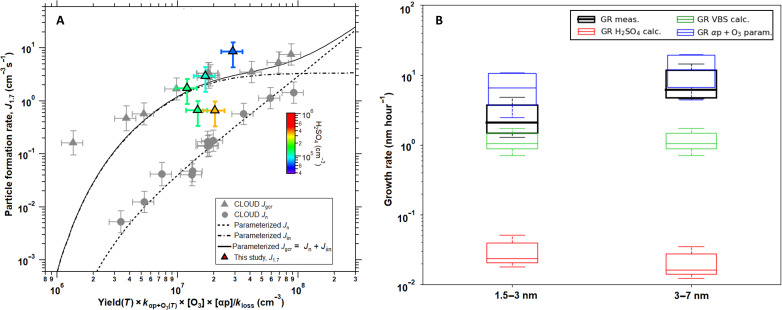
Atmospheric particle formation rates and GRs for the clustering with growth events in Siikaneva. (**A**) Particle formation rates at 1.7 nm (*J*_1.7_) versus the total HOM production from reacted α-pinene (αp). The *x* axis has taken into account yield (*T*); the HOM yield from the ozonolysis of α-pinene at the corresponding temperature (*74*); the reaction rate coefficient of α-pinene with O_3_, k_αp+O3(T)_ (International Union of Pure and Applied Chemistry; https://iupac.aeris-data.fr/en/home/); and the HOM total loss rate (*k*_loss_) (*7*), which is the CS level for the field data (*15*). The color scale shows the corresponding ambient sulfuric acid concentration. Please note that only five clustering with growth events had complete particle and precursor data. Experimental pure biogenic nucleation rates at 1.7 nm (*J*_1.7_) for neutral *J*_n_ (circle) and galactic cosmic ray *J*_gcr_ (triangle) from Kirkby *et al.* (*7*) are indicated by gray markers, and corresponding parametrizations for neutral *J*_n_ (dashed), galactic cosmic ray *J*_gcr_ (solid), and ion-induced nucleation (*J*_iin_ = *J*_gcr_ − *J*_n_; dot-dashed) by black lines, with the bars indicating 1σ total errors (*7*). (**B**) Comparison of measured GR with GR calculated by assuming sulfuric acid contribution to growth (*31*) (red) or by also assuming a contribution from α-pinene ozonolysis reactivity (*34*) (blue) or directly from measured HOM via VBS calculation (*33*) (green). Lower and upper quartile of the boxes represent 25th and 75th percentiles, respectively.

In Fig. 3A, particle formation rates at 1.7 nm (*J*_1.7_) are plotted versus the total HOM production from reacted α-pinene for the clustering with growth events to compare to the experimental pure biogenic nucleation results from CLOUD chamber at CERN (*7*). As we can see, the measured *J*_1.7_ (0.7 to 8.6 cm^−3^ s^−1^) for these clustering with growth events are close to the pure biogenic *J*_1.7_ measured at CLOUD at similar HOM concentrations, particularly the galactic cosmic ray *J*_gcr_, indicating the important role of ions in facilitating the nucleation in Siikaneva (*30*). Together, these results indicate that it is plausible that pure biogenic HOM initiated the observed nucleation in Siikaneva.

Furthermore, Fig. 3B shows that the measured growth rates (GRs) increased from 2.1 nm hour^−1^ in the 1.5 to 3 nm in size range to 6.2 nm hour^−1^ in the 3 to 7 nm in size range. These values are much higher than the GR calculated by assuming only the condensation of sulfuric acid (<0.03 nm hour^−1^) (*31*) and thus suggest a dominant contribution of other vapors such as HOM. For the smaller size range (1.5 to 3 nm), the GR calculated from HOM using a volatility basis set (VBS) (*32*) were, on average, a factor of ~2 lower than the observed values, which is within the uncertainty range of the approach (*33*). At the larger size range (3 to 7 nm), the VBS approach underestimated the observed growth by a factor of ~6, likely as a result of underestimation of low volatility organic compounds (LVOCs; see details in Materials and Methods) due to limited sensitivity of CI-APi-TOF toward LVOC (*34*). However, we obtained closure within a factor of ~2 for the GR in the 3 to 7 nm in size range using a biogenic GR parametrization based on α-pinene ozonolysis reactivity, which better accounts for the contribution of LVOC (*34*). This α-pinene ozonolysis reactivity-based parametrization overestimated the observed GR by a factor of ~3 for the smaller size range (1.5 to 3 nm). These discrepancies of two to three between observed and parametrized GR are within uncertainties, specifically in the context that ambient biogenic VOC in Siikaneva are not pure α-pinene (Fig. 1D and fig. S2) (*21*).

Together, our results suggest that HOM of biogenic origin are the dominant contributor to the particle growth of the nighttime NPF events at this measurement site. For the clustering without growth events, the potential GR calculated from vapor concentrations were on average a factor of ~2 lower than those for the clustering with growth events, for both methods and size ranges (fig. S10). The absence of observed further growth is, at least partially, due to the lower survival probability of small particles [depending exponentially on coagulation sink (CoagS)/GR, where CS can act as a proxy for CoagS] (*35*) caused by higher CS values for clustering without growth events (figs. S9C and S10).

### Particle formation mechanism

**Fig. 4.**
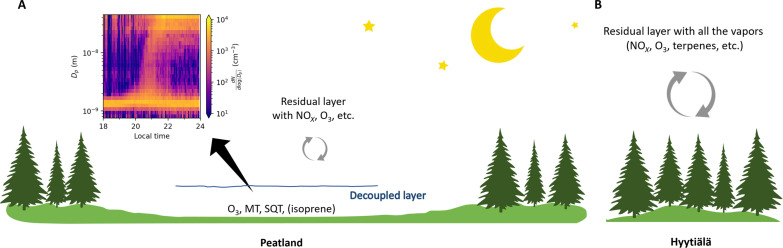
Schematic for nighttime atmosphere in Siikaneva peatland and the nearby Hyytiälä boreal forest. (**A**) “Pre-industrial-like atmosphere” hidden inside the air pocket, i.e., a decoupled layer (up to few meters high) over Siikaneva peatland. The stable and shallow decoupled layer isolates the peatland from outside anthropogenic pollutants (e.g., NO*_x_*) at night, with initial gases (e.g., O_3_) or anthropogenic pollutants (e.g., NO*_x_*) inside largely depleted through dry deposition onto the wet peatland surface within the shallow decoupled layer. Meanwhile all peatland emissions (e.g., monoterpenes, sesquiterpenes, and isoprene) are trapped and concentrated inside the air pocket. This favors pure biogenic HOM-induced NPF during the night over the peatland. Insert shows the size evolution of the positive ions measured by NAIS on the night of 20 May 2016. (**B**) “Present-day atmosphere” with a boundary layer height of few hundreds of meters in the nearby (~5 km away) Hyytiälä boreal forest (*75*). The rough surface or complex terrain enhances mixing, which results in dilution of CHO HOM dimer concentrations so that pure biogenic NPF cannot be triggered.

To summarize, we provide unambiguous evidence of pure biogenic HOM-induced NPF, i.e., nucleation followed by growth to larger sizes (e.g., at least to Aitken mode), during the nighttime in the Siikaneva peatland (Fig. 4A). These nighttime NPF events were characterized by a stable and shallow decoupled layer of air (*15*) associated with a strong near-surface cooling and reduced shear and turbulence due to flat topography of the peatland surface (*36*). This contrasts to daytime NPF involving sulfuric acid (see an example for 3 May in figs. S2 and S4) in an atmosphere well mixed with pollutants (e.g., NO*_x_*, SO_2_, anthropogenic HOM, etc.), which is the case for the daytime NPF in the nearby Hyytiälä forest (*3*, *15*). The nighttime air pocket (i.e., decoupled layer) above Siikaneva isolated the peatland from replenishment of the outside anthropogenic pollutants (e.g., NO*_x_*) (*37*), and meanwhile, many initial vapors or anthropogenic pollutants inside were depleted through dry deposition onto the wet peatland surface within the shallow decoupled layer (see also the example in the nearby Hyytiälä forest in fig. S3) (*22*–*24*). The low concentrations of CHON HOM dimers—markers for the NO_3_ chemistry (*18*)—indicate that NO*_x_* levels are very low in Siikaneva at decoupled event nights. Although small amounts of NO_3_ radicals were possibly still present at the beginning of the decoupled event nights and responsible for some CHON HOM dimer formation (see Fig. 1 and fig. S2), no more NO_3_ radicals and CHON HOM dimers were produced, in contrast to coupled nonevent nights (e.g., Fig. 2). Even during the coupled nonevent nights, NO*_x_* was estimated to be only 0.4 ppbv (parts per billion by volume), assuming a well-mixed atmosphere under turbulent conditions, based on the NO*_x_* measurements in the nearby Hyytiälä forest. Furthermore, the air pocket also trapped and concentrated all peatland emissions, including terpenes, leading to the formation and clustering of pure biogenic HOM over the peatland.

The nighttime NPF observed in Siikaneva mimics the pre-industrial atmosphere, where no anthropogenic influence is apparent. This nocturnal phenomenon in Siikaneva was driven by O_3_ but not by NO_3_ radicals or OH radicals (see low radiation levels in Fig. 1B and fig. S2), although OH radicals could be important for daytime photochemistry driven NPF during the pre-industrial era. Although O_3_ was lower during decoupled event nights (<25 ppbv) than during the daytime (see Fig. 1C and fig. S2) as a result of dry deposition and chemical loss (*22*, *24*), O_3_ chemistry was strong enough for the formation of CHO HOM dimers responsible for the nighttime NPF. Besides, low O_3_ surface concentration also mimics pre-industrial levels as O_3_ is believed to have been much lower (<10 ppbv) than today (*22*, *38*), although the O_3_ levels in the air pocket of Siikaneva peatland were still higher than those during pre-industrial era.

This study indicates that favorable meteorology is required for NPF observation under pristine-like conditions in the present-day atmosphere. This is true even in boreal forests such as Hyytiälä with large monoterpene emissions, where daytime NPF involves sulfuric acid formed from anthropogenic SO_2_ emissions, while nighttime newly formed particles do not grow to larger sizes (e.g., to the Aitken mode; see Fig. 4B) (*14*). For example, the rough surface or complex terrain with even a gentle hill could enhance mixing [with a decoupling process happening only ~19% of the nights in the nearby Hyytiälä forest (*39*)], which would result in dilution of emitted vapors (see a clustering without growth example for Hyytiälä in fig. S11). In addition, higher NO*_x_* (*17*, *40*, *41*) and NO_3_ (*18*) levels tend to suppress nucleation and early growth in the present-day atmosphere. Previous studies have shown that anthropogenic pollutants such as NO*_x_* hinder NPF due to the inhibition of the formation of large HOM dimers (*3*, *17*), which are less volatile and thus critical for “pure biogenic” nucleation (*7*). Instead of forming HOM dimers with larger molecular weight and additional functional groups, the NO*_x_*-perturbed condition favors the production of CHON HOM monomers (*17*). However, not all HOM dimers are low volatile enough to drive the NPF. NO_3_ radicals [derived from NO*_x_* (*16*)] are also found to suppress the α-pinene HOM-induced NPF as the NO_3_ radical chemistry forms higher volatility CHON HOM dimers. These highly functionalized CHON HOM dimers with large molecular weight are less oxidized and thus too volatile (compared to CHO HOM dimers) to initiate nucleation (*18*). Whether CHON HOM dimers formed in environments dominated by other monoterpenes such as Δ-3-carene could drive the ambient nucleation alone needs more field observations (*42*). The results indicate that in the present-day global atmosphere with ubiquitous anthropogenic pollution, it is not only pre-existing particles (enhancing condensation/CS or CoagS) that impedes NPF by acting as a sink for nucleating vapors or newly formed particles (*35*) but also the NO*_x_*-related chemistry that would suppress pure biogenic HOM-driven NPF.

## DISCUSSION

Our results from Siikaneva peatland show that truly pristine environments do exist in the present-day atmosphere, as previous modeling results have already shown (*43*). Under suitable environmental conditions with high enough levels of biogenic vapors, pure biogenic nucleation can produce particles that grow to larger sizes, which may contribute to regional aerosol number budget and pose potential subsequent impacts on climate, e.g., for areas dominated by peatlands and other locations with flat topography. However, the observed pure biogenic particles (at least 42-nm particle) formed at night within the shallow surface layer with limited area/volume above Siikaneva likely represent only a small fraction of the ambient CCN, particularly in the present-day atmosphere after they are mixed upward into the planetary boundary layer during the daytime. However, considering the large spatial coverage of peatlands (~4 million km^2^ north of 30°N and 0.5 million km^2^ north of 50°N) (*15*), pure biogenic particles formed from peatlands from nighttime NPF (as well as daytime photochemistry driven NPF) may be an important source of climatically relevant CCN particularly during the pre-industrial era, which requires more dedicated simulation studies for peatlands in the future.

Pure biogenic HOM-induced nucleation we observed in the air pocket of Siikaneva peatland could be one of those similar to the nighttime NPF in the pre-industrial atmosphere. It could be the nucleation mechanism for the frequent nighttime NPF in other environments as well such as in the subboreal forests of North America (*10*), and thus, nighttime NPF in conditions similar to the pre-industrial atmosphere might be a widespread and frequent phenomenon in the present day, which calls for more dedicated future studies in different environments. Besides, these biogenic (mono)terpenes from vegetation are not the only natural vapors capable of forming particles. Sulfuric acid and ammonia/amines from non-anthropogenic sources [e.g., sulfuric acid from the oxidation of marine dimethyl sulfide or volcanic eruptions (*28*) and ammonia/amines emitted by vegetation, ocean or animal excreta (*44*)] could be precursors for pre-industrial NPF as well (*45*). Iodine, emitted by algae, can be another important biogenic precursor for pre-industrial NPF, as iodine oxoacids have been shown to be able to independently form new particles rapidly, competing with sulfuric acid(−ammonia) particle formation in pristine coastal/polar environments (*4*). However, careful consideration needs to be taken to differentiate the biogenic and anthropogenic origins of these precursors when NPF is observed in the present anthropogenically perturbed atmosphere (*3*, *7*, *28*, *46*). Furthermore, complex interactions between biogenic and anthropogenic vapors in particle formation are still insufficiently understood. Future studies of environments like Siikaneva could advance our knowledge on pre-industrial aerosol as it affects the baseline for calculating aerosol forcing, the variance of which has been found to arise more from the uncertainties in natural emissions rather than anthropogenic emissions (*47*).

Our results may also provide hints for understanding NPF in the future. Because of air pollution mitigation, the future atmosphere may resemble more the pre-industrial atmosphere; i.e., the role of acid-base clustering mechanism may start to be replaced (at least partially) by pure biogenic organic vapors (*48*). The question arises whether this will also change the future NPF frequency and intensity. This has been traditionally thought of as a competition between decreasing CS levels (which favors NPF) (*35*) and sulfur emissions (which reduces NPF) (*49*) due to decreasing fossil fuel consumption and increasing biogenic emissions caused by higher temperatures (which favors NPF) (*50*). In addition, decreasing anthropogenic NO*_x_* emissions as a result of environmental regulations, and thus all NO*_x_*-related chemistry, could also favor pure biogenic HOM-induced NPF (*17*, *18*). It is unclear which of these factors would have the dominating effect on the future NPF chemistry and frequency. The present work is limited to nighttime O_3_-induced NPF, whereas daytime photochemistry-driven NPF dominates almost everywhere in the present-day atmosphere (*5*). While it might be possible that the role of pure biogenic HOM-induced nighttime NPF will gradually increase, it is still important to investigate potential biogenic HOM-induced daytime NPF in air unpolluted by SO_2_ or NO*_x_*. To address these questions, more detailed and realistic parametrizations must be developed, followed by model simulations for a better understanding of the characteristics (including frequency, intensity, and timing) of future NPF and subsequent effects on CCN and aerosol-cloud forcing. Our study thus underlines the significance of pre-industrial aerosol research, which has been a relatively neglected area of climate science but nevertheless is important for understanding NPF and aerosol in the future atmosphere.

## MATERIALS AND METHODS

### Instrumental setup

The data were part of the campaign performed between 10 March and 20 June 2016, at Siikaneva peatland in southern Finland. The pristine peatland Siikaneva is located in southern Finland [61°49′59.4″N, 24°11′32.4″E, 162 m a.s.l. (above sea level)]. It is a class II ecosystem Integrated Carbon Observation System (ICOS) station, which is ~5 km west to the Station for Measuring Ecosystem–Atmosphere Relations (SMEAR) II station, Hyytiälä (*51*) and ~60 km NE to the nearest big city, Tampere, with more than 200,000 inhabitants (*52*). Hyytiälä site has complex terrain and is dominated by Scots pine (*Pinus sylvestris*) with dominating monoterpene emissions (*53*, *54*). In contrast, this peatland is characterized with relatively flat topography. It is a 9000-year-old minerotrophic peat and mostly covered with fens with a number of vegetation communities and some surface patterning featuring drier hummocks and wetter lawns (*15*), emitting large amounts of terpenes (dominated by isoprene, α-pinene, etc.) (*21*).

The molecular composition of both ambient positively charged clusters and negatively charged clusters was analyzed with an APi-TOF mass spectrometer (TOFWERK AG.). Air ions were sampled via a 10-mm stainless steel tube and a total sample flow of 6 liter min^−1^ (only 0.8 liter min^−1^ into the instrument through a critical orifice) and then guided by two quadrupoles and an ion lens to the TOF mass spectrometer.

The molecular composition of ambient neutral clusters as well as the concentrations of sulfuric acid and highly oxygenated organic molecules (HOM) were measured with a CI-APi-TOF (TOFWERK AG.) using NO_3_^−^ as the reagent ion. Air was sampled through a ¾-inch stainless steel tube and a flow rate of 10 liter min^−1^. An x-ray charger (4.9 kV; Hamamatsu Photonics K.K.) was used to ionize the reagent nitric acid vapor in an air flow of 6 cm^−3^ min^−1^, entraining a sheath flow rate of 30 liter min^−1^. Nitrate ions are then guided to react with the analyte molecules in the sample flow. After being charged, the analyte molecules are guided by two quadrupoles and an ion lens assembly to the TOF mass analyzer. Data were analyzed with the software package, “tofTools” [developed by Junninen *et al.* (*55*)], which run in the MATLAB environment (MathWorks Inc., USA). The quantification of sulfuric acid and HOM was calculated (*56*) as in Eq. 1

$$\left[\text{comp}\right]=\frac{\text{comp}\left({\text{NO}}_{3}^{-}\right)}{\sum_{i\;=\;0}^{2}{\left(\text{HN}O_{3}^{-}\right)}_{i}\left(NO_{3}^{-}\right)}\times C_{NO_{3}^{-}}$$ (1)

where [comp] is the concentration (unit: cm^−3^) of the gaseous compound (obtained from high-resolution fitting of each nominal mass) to be quantified; the numerators on the right-hand side are its detected signal clustered with nitrate, and the denominators are the sum of the reagent ion signals; $C_{NO_{3}^{-}}$ is the calibration factor representing the sensitivity of gaseous compound. $C_{NO_{3}^{-}}$ for sulfuric acid [H_2_SO_4_, compound representing the kinetic limit sensitivity (*57*, *58*)] was determined (*15*) to be 5 × 10^10^ cm^−3^. With the maximum sensitivity applied, the HOM concentrations therefore represent a lower limit. The uncertainties in the measured HOM concentrations using calibration factors for H_2_SO_4_ have been reported to be ±50% (*59*) or a factor of 2 (*60*).

VOC concentrations including isoprene, monoterpenes, and sesquiterpenes were measured with a PTR-TOF mass spectrometer 8000 (Ionicon Analytik GmbH) (*61*). The drift tube, where the VOCs are protonated by H_3_O^+^ ions, was heated to 60°C and had an operational pressure of 2.3 mbar. Together with the applied drift voltage of 600 V, this led to an *E*/*N* (*E* is the electric field strength, and *N* is the number density in the reaction cell) of 130 Td (Townsend; 10^−21^ V m^2^). The ambient air was pumped with 20 liter min^−1^ as a carrier flow, through a 10-m-long polytetrafluoroethylene tube with an inner diameter of 8 mm. The PTR-TOF was connected with a three way valve (type: 6606 with ethylene tetrafluoroethylene, Bürkert GmbH & Co. KG, Germany) to this inlet taking a sample air of 0.8 to 1.0 liter min^−1^, while the other port of the valve was connected to a custom build catalytic converter. The generated VOC-free zero air was used to measure the instrumental background automatically three times a day. During the measurement period, the instrument was calibrated seven times using diluted calibration gas of around 6 ppbv (Apel Riemer Environmental Inc., USA), which included among other VOCs also isoprene and α-pinene. Detailed information about the calibration setup, data analysis, and the bulk sensitivity approach to gain the sensitivity for the sesquiterpenes is described in (*62*).

Number concentrations of particles with a mobility diameter larger than 2.5 nm were recorded with a butanol-based condensation particle counter (CPC3776, TSI Inc.). A neutral cluster and air ion spectrometer (NAIS, Airel Ltd.) was used to measure the number size distribution of atmospheric ions and neutral particles with a mobility diameter of 0.8 to 42 nm (*63*). The instrument switched periodically among different modes (ion, particle, and offset), with one cycle typically lasting 150 s. In the ion mode, only ions and naturally charged particles were detected. In the particle mode, particles were charged using a corona charger before entering the detector. During the offset mode, the instrument removed all particles from the sample flow to determine the offset signal. For the number size distribution measurement of sub–3-nm particles, an Airmodus A11 nano Condensation Nucleus Counter (nCNC), consisting of an A10 particle size magnifier (PSM, Airmodus Ltd.) (*64*) coupled with a A20 CPC was deployed. Core-sampling similar as in Kangasluoma *et al.* (*65*) was adopted using a sampling line of 40-cm-long stainless steel tube with an inner diameter of 8 mm. The data inversion was performed using Gaussian-shaped kernel functions described in (*66*).

Air temperature and relative humidity were measured with a Rotronic HC2 sensor (Rotronic AG.). Global radiation was detected by a Kipp & Zonen CNR4 radiometer (OTT HydroMet B.V.). Wind speed and direction were measured with a HS-50 anemometer (Gill Instruments Ltd.).

All instrumentations were set up in a small measurement container. Sampling inlets were located at heights of approximately 1.5 m and 3 m a.g.l. (above ground level). The CI-APi-TOF, APi-TOF, NAIS, PSM, and O_3_ measurements were conducted with the inlet at 1.5 m, while all the meteorological, wind speed, wind direction, CO_2_, CH_4_, and VOC data were obtained with the inlet at 3 m. Turbulence (i.e., friction velocity) and concentrations of CO_2_ at the 3-m height were used to diagnose the boundary layer state: Low turbulence and elevated CO_2_ concentration indicated decoupling; and CH_4_ concentration at the 3-m height, in combination with a simple box model, allowed to determine the height of the lowest decoupled layer: Low CH_4_ concentration indicated decoupling below 3 m, whereas high CH_4_ concentration in together with the box model simulations found the height of decoupling to be 4 m (*15*). Detailed description of the instrumentation and diagnosis of the surface layer state and decoupling can be found in (*15*) All data are reported in eastern European time (UTC +2).

### Determination of particle formation rates and GRs

The neutral particle formation rates *J_dp_* and ion formation rates $J_{\mathrm{dp}}^{\pm }$ at size *dp* were calculated for the nighttime NPF events according to the approach by Kulmala *et al.* (*67*) as in Eqs. 2 and 3

$$J_{\mathrm{dp}}=\frac{{\mathrm{dN}}_{\mathrm{dp}}}{\mathrm{dt}}+{\text{CoagS}}_{\mathrm{dp}}\times N_{\mathrm{dp}}+\frac{\text{GR}}{∆\mathrm{dp}}\times N_{\mathrm{dp}}+S_{\text{losses}}$$ (2)

where $\frac{{\mathrm{dN}}_{\mathrm{dp}}}{\mathrm{dt}}$ is the time evolution of particle number concentration *N_dp_*, CoagS*_dp_* is the CoagS of particles in the size range [*dp*, *dp* + Δ*dp*], GR is their growth rate, and *S*_losses_ includes additional losses.

$$\begin{array}{c} J_{\mathrm{dp}}^{\pm }=\frac{{\mathrm{dN}}_{\mathrm{dp}}^{\pm }}{\mathrm{dt}}+{\text{CoagS}}_{\mathrm{dp}}\times N_{\mathrm{dp}}^{\pm }+\frac{\text{GR}}{∆\mathrm{dp}}\\ \times N_{\mathrm{dp}}^{\pm }+\alpha \times N_{\mathrm{dp}}^{\pm }\times N_{<\mathrm{dp}}^{\mp }-\chi \times N_{\mathrm{dp}}\times N_{<\mathrm{dp}}^{\pm } \end{array}$$ (3)

where $\alpha \times N_{\mathrm{dp}}^{\pm }\times N_{<\mathrm{dp}}^{\mp }$ represents ion-ion recombination, $\chi \times N_{\mathrm{dp}}\times N_{<\mathrm{dp}}^{\pm }$ is charging of neutral particles by smaller ions, α is ion-ion recombination coefficient, χ is ion-aerosol attachment coefficient, and subscript <*dp* refers to all particles smaller than *dp* in diameter. We used the NAIS to calculate particle formation rates at 3 nm (using the 3- to 7-nm size bin and Eq. 2, where the NAIS shows consistent total number concentrations with other instruments) and scaled it to 1.7 nm using the adjusted Kerminen and Kulmala equation (*68*), which shows good validity in the nearby Hyytiälä forest (*69*).

The ion and particle GRs were calculated from the NAIS data in the 1.5- to 3-nm and 3- to 7-nm size ranges. The CS was calculated using the differential mobility particle sizer (DMPS) data in the nearby (~5 km away) Hyytiälä forest (*15*). We assume that the larger particles that mostly contribute to the CS have similar size distributions in Hyytiälä and in Siikaneva. This has already been justified in a previous paper by Junninen *et al.* (*15*).

GRs were determined from the NAIS data via the appearance time method. For the NAIS data, the GRs of all particles (NAIS particle mode) and ions (NAIS ion mode) were determined for the 1.5- to 3-nm and 3- to 7-nm size range by a linear fit to the appearance time of the particles or ions in different size ranges. Further details on this method and uncertainties can be found in (*66*) and (*34*).

The NAIS data were used to classify the nucleation events based on (*70*) and (*71*):

Clustering with growth event: Appearance of a growing mode of particles that starts from ~2 nm and continues to the Aitken mode (i.e., tens of nanometers) usually for several hours. Number concentrations are high compared to the background, and growth and formation rates can be determined.

Clustering without growth event: Increased particle number concentrations in the ~2- to 4-nm size range that last more than an hour. However, growth to Aitken mode (i.e., tens of nanometers) is not observed.

Nonevents: Neither small particles nor growth are observed.

### Determination of modeled GRs

The GR (nm hour^−1^) explained by only assuming sulfuric acid contributions to particle growth was calculated using the approach by Stolzenburg *et al.* (*31*), assuming kinetic growth (i.e., no re-evaporation of condensed sulfuric acid) and dipole-dipole interactions enhancing the collisions.

The GR (nm hour^−1^) explained by also assuming a contribution of α-pinene ozonolysis reactivity using the parametrization by Stolzenburg *et al.* (*34*) as in Eq. 4 was used, which represents pure organic growth resulting from biogenic emissions

$${\text{GR}}_{\alpha p+O3\;\text{param}.}=m\left(T,d_{p}\right)\times {\left[k\left(T\right)\times \alpha p\times O_{3}\right]}^{q}$$ (4)

where *m*(15°C, 1.5 to 3 nm) = 1.6 × 10^−7^, *m*(15°C, 3 to 7 nm) = 2.9 × 10^−7^ are the mean values from the 5° and 25°C results from (*34*) and *q* = 1.21.

The GR calculated using the volatility distribution of the observed HOM was obtained following the approach of Stolzenburg *et al.* (*33*), where the volatility [i.e., the effective saturation mass concentrations (*C*_sat_)] of each molecule ion observed with the CI-APi-TOF was calculated following the parametrization from (*34*) as in Eq. 5 but without a differentiation between monomers and dimers, as they were not well-defined in the Siikaneva HOM mixture.

$$\begin{array}{c} {\text{log}}_{10}C_{\text{sat}}=\left(n_{C}^{0}-n_{C}\right)\times b_{C}-\left(n_{O}-3\times n_{N}\right)\times b_{O}-2\\ \times \frac{n_{C}\times \left(n_{O}-3\times n_{N}\right)}{\left(n_{C}+n_{O}-3\times n_{N}\right)}\times b_{\text{CO}}-n_{N}\times b_{N} \end{array}$$ (5)

where *n*_C_, *n*_O_, and *n*_N_ are the number of carbon, oxygen, and nitrogen atoms in the organic compound, respectively; $n_{C}^{0}$ = 25; *b*_C_ = 0.475, *b*_O_ = 1.4, *b*_N_ = 2.5, and *b*_CO_ = −0.3.

Molecular ion signals were then grouped into volatility bins at 300 K spaced by one order of magnitude in *C*_sat_ and then shifted to the corresponding ambient temperature of the clustering event. Organic compounds with *C*_sat_ lower than 10^–8.5^ μg m^−3^, between 10^–8.5^ and 10^–4.5^ μg m^−3^, between 10^–4.5^ and 10^–0.5^ μg m^−3^, between 10^–0.5^ and 10^2.5^ μg m^−3^, between 10^2.5^ and 10^6.5^ μg m^−3^, and higher than 10^6.5^ μg m^−3^ are termed ULVOC, ELVOC, LVOC, SVOC, IVOC, and VOC, respectively (*72*, *73*). Mass flux to a monodisperse particle population was then numerically solved using the code by Stolzenburg *et al.* (*33*), accounting for curvature and solution effects on the saturation vapor pressures.
