# Supplementary material for: Potential pre-industrial–like new particle formation induced by pure biogenic organic vapors in Finnish peatland
Source: Sci Adv. 2024 Apr 3;10(14):eadm9191. doi: 10.1126/sciadv.adm9191 (PMC10990286; doi:10.1126/sciadv.adm9191)
Supplement: Supplementary file 1 — Figs. S1 to S11 Table S1 [file sciadv.adm9191_sm.pdf]

Science Advances
AAAS

Supplementary Materials for

Potential pre-industrial–like new particle formation induced by pure biogenic organic vapors in Finnish peatland

Wei Huang *et al.*

Corresponding author: Federico Bianchi, federico.bianchi@helsinki.fi

Sci. Adv. **10**, eadm9191 (2024)
DOI: 10.1126/sciadv.adm9191

This PDF file includes:

Figs. S1 to S11
Table S1

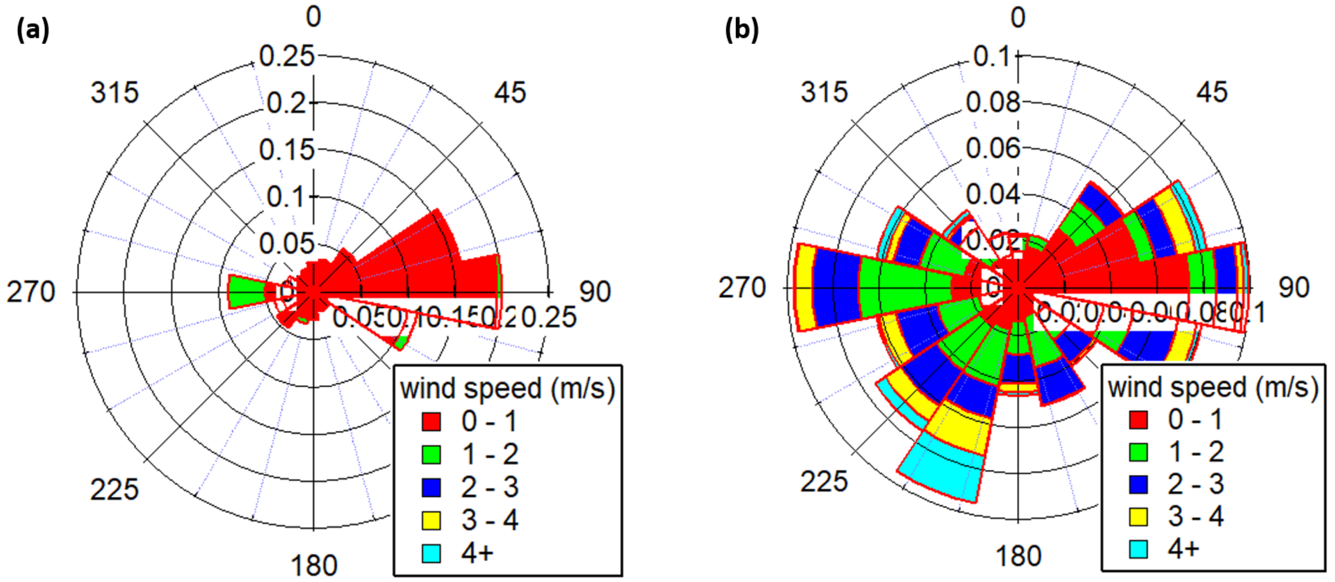

Fig. S1. **Wind rose plots in Siikaneva during the measurement period. (a)** Decoupled nights and **(b)** all nights. Please note that with very low wind speed for decoupled nights, wind direction in (a) is not an indication for the air mass.

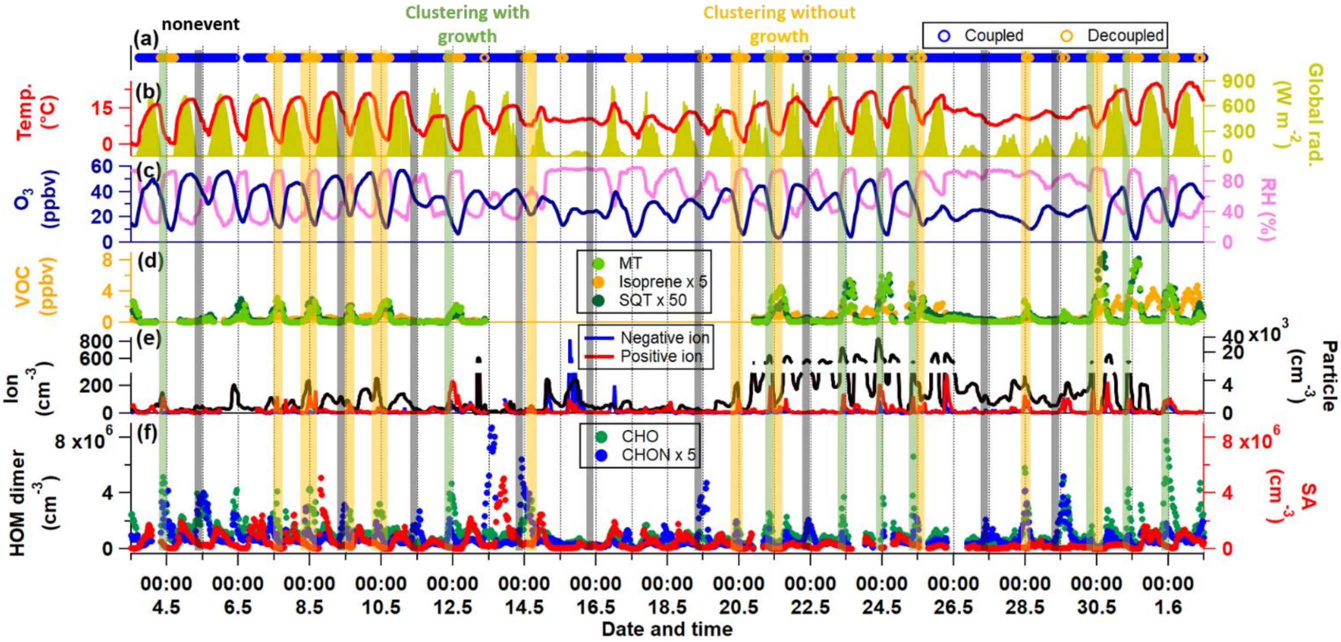

Fig. S2. Nighttime events observed in Siikaneva. Data are from May 3 to June 1, 2016, during which we observed 9 clustering with growth events (shaded in green), 10 clustering without growth events (shaded in orange), and 9 nonevents (shaded in gray; see more details in Table S1). **(a)** Surface layer state; **(b)** temperature and global radiation; **(c)** mixing ratios of O_3 and relative humidity (RH); **(d)** mixing ratios of isoprene, monoterpenes (MT), and sesquiterpenes (SQT) measured with a proton transfer time-of-flight mass spectrometer (PTR-TOF); **(e)** number concentrations of intermediate ions (2−4 nm) and atmospheric clusters (1.1−2.5 nm), measured with a neutral cluster and air ion spectrometer (NAIS) and a particle size magnifier (PSM), respectively; and **(f)** HOM dimer and sulfuric acid (SA) concentration measured with the CI-APi-TOF. These neutral gas-phase precursor vapors could form clusters, and grow into nanoparticles after stabilization and activation (*5*). All data are reported in eastern European time (UTC+2).

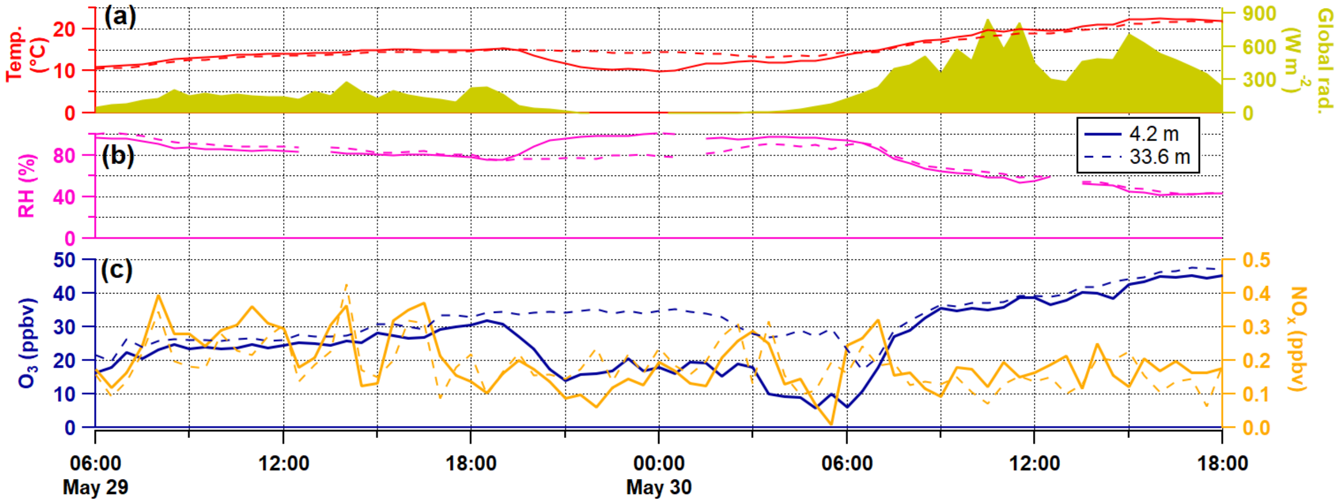

Fig. S3. A decoupling example observed in the nearby boreal forest in Hyytiälä. Data are from the night of May 29, 2016 for **(a)** temperature and global radiation, **(b)** relative humidity (RH), and **(c)** mixing ratios of O_3 and NO_x, near the ground surface at 4.2 m (in solid lines) and above the forest canopy at 33.6 m (in dashed lines). We can see near-surface cooling and ozone depletion starting around 19:30 at 4.2 m, and NO_x at 4.2 m also decreased soon after. In addition to the chemical loss, the trace gas decrease is also due to the dry deposition to the wet ground surface (RH reaching almost 100 %) (*23, 24*).

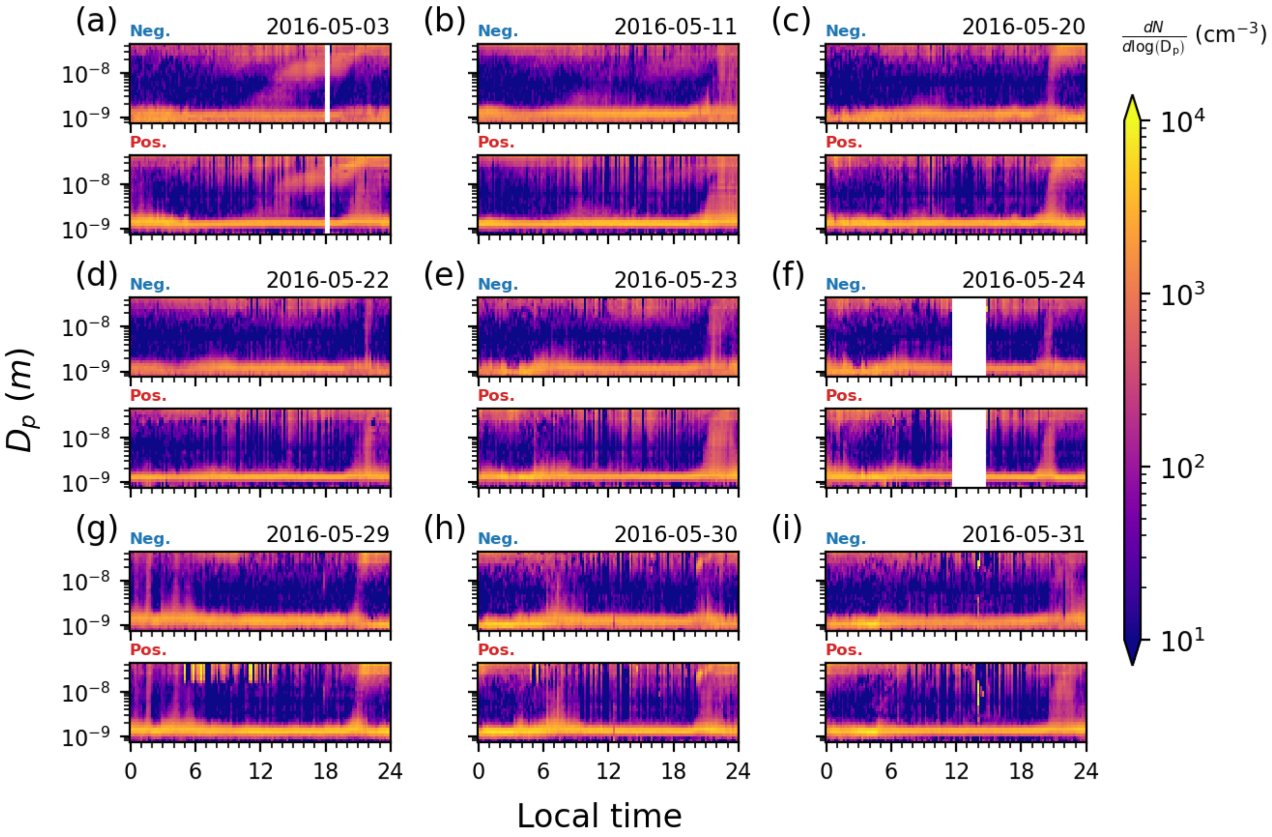

Fig. S4. Nighttime clustering with growth events observed by NAIS in Siikaneva. Data are from the size evolution of positive and negative ions for the 9 clustering with growth events on **(a)** May 3, **(b)** May 11, **(c)** May 20, **(d)** May 22, **(e)** May 23, **(f)** May 24, **(g)** May 29, **(h)** May 30, and **(i)** May 31, 2016. The color scale shows the concentration of ions. Some nighttime clustering without growth events were also seen for some late night or early morning periods (e.g., May 20 00:00−05:00 and May 29 22:00−06:00). Please note that some daytime clustering events were observed as well (e.g., May 3 10:00−16:00).

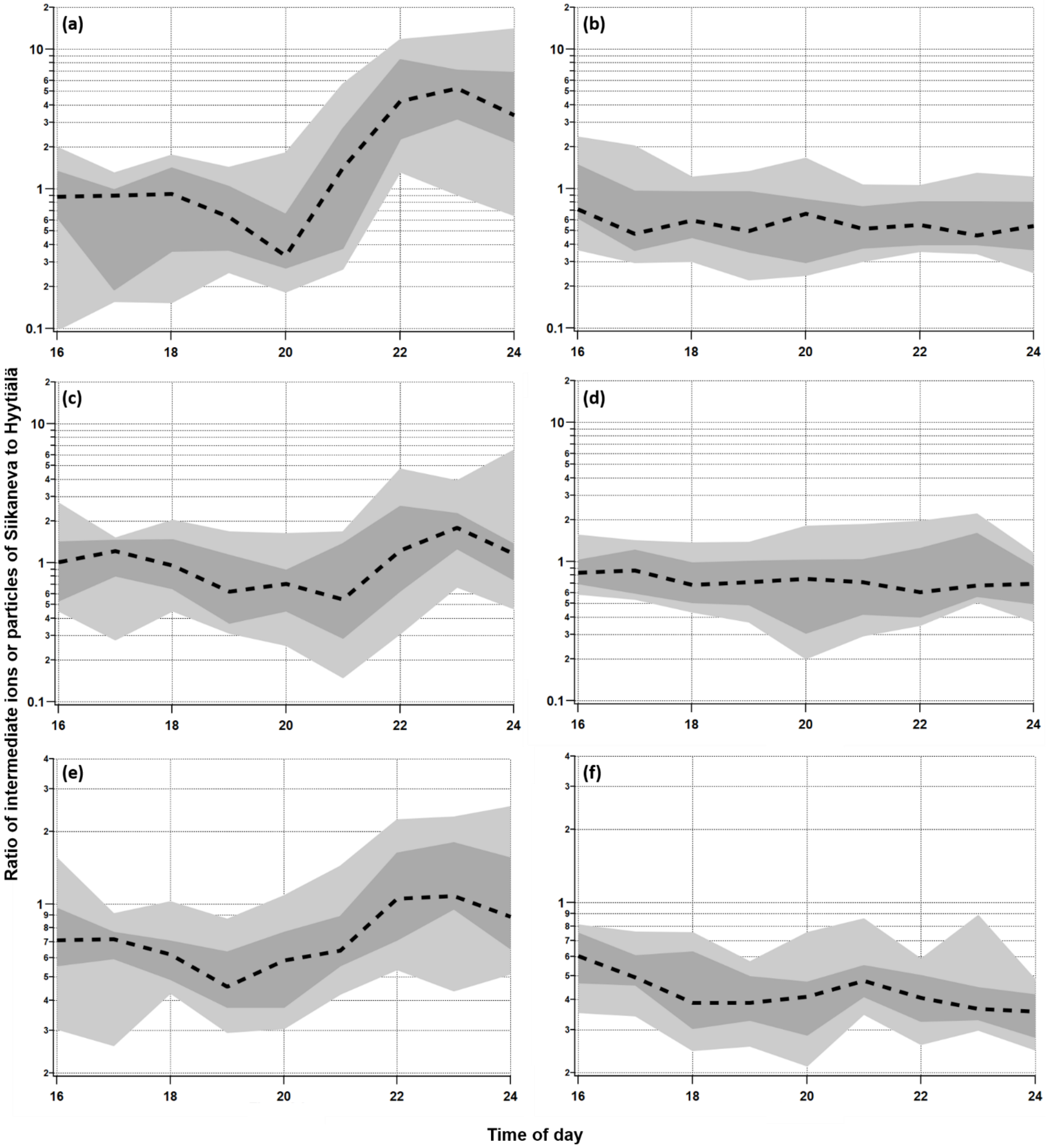

Fig. S5. Origin of intermediate ions and particles measured by NAIS for nighttime clustering with growth events in Siikaneva. Ratio of intermediate positive ions (2–4 nm) of Siikaneva to the nearby forest in Hyytiälä for clustering with growth events **(a)** and nonevents **(b)**. Ratio of intermediate negative ions (2–4 nm) of Siikaneva to Hyytiälä for clustering with growth events **(c)** and nonevents **(d)**. Ratio of total particles (2.5–3 nm) of Siikaneva to Hyytiälä for clustering with growth events **(e)** and nonevents **(f)**. Median values are shown in dashed black lines, with the 10th, 25th, 75th, and 90th percentiles shown in the shaded area. Higher ratios of intermediate ions and small particles for the nights of clustering with growth events in Siikaneva suggest that NPF in Siikaneva was a local phenomenon and the origin of intermediate ions or small particles was from Siikaneva peatland but not from the nearby Hyytiälä forest.

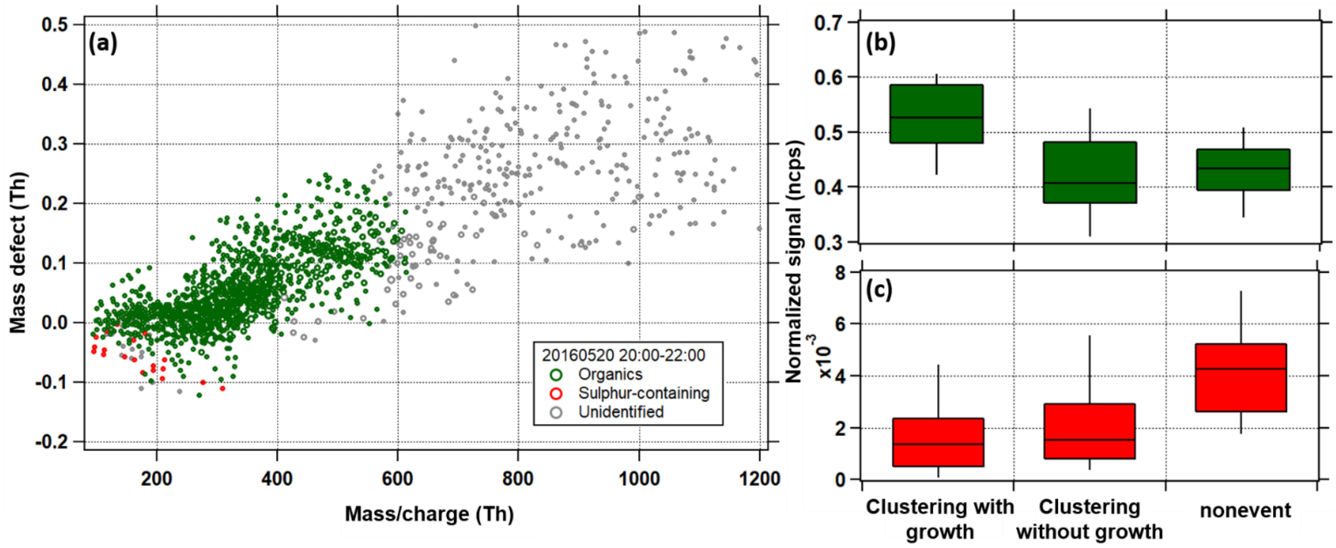

Fig. S6. Negative ion composition in Siikaneva measured with APi-TOF operated in negative mode. **(a)** Negative ion mass defect plot for a clustering with growth event. The size of the circles is proportional to the intensity of the signal. Organics would have a positive mass defect while sulfur-containing species would possess a negative mass defect. Normalized signal of **(b)** total negatively charged HOM (mass-to-charge >400 Th), and **(c)** sulfuric acid ion for clustering with growth events, clustering without growth events, and nonevents. Lower and upper quartile of the boxes represent 25th and 75th percentiles, respectively. While sulfuric acid ion signal was lowest for the clustering with growth events, negatively charged HOM exhibited the highest signal. Besides, large charged sulfuric acid clusters, such as sulfuric acid tetramer which is detected only in the presence of ammonia (*3*), or sulfuric acid clusters with ammonia and dimethylamine (DMA) (*2*) were absent in the mass defect plot during clustering events.

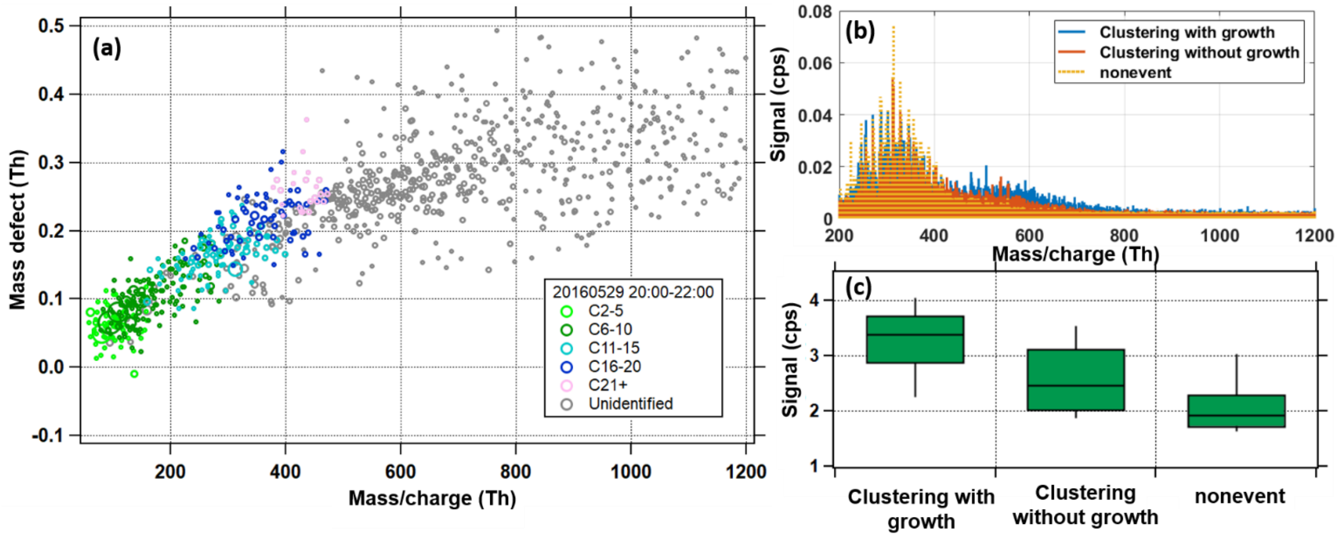

Fig. S7. Positive ion composition in Siikaneva measured with APi-TOF operated in positive mode. **(a)** Positive ion mass defect plot for a clustering with growth event. The size of the circles is proportional to the intensity of the signal. **(b)** Mass spectra of positive ions, and **(c)** signal of total large positive ions (mass-to-charge ratio >500 Th) for clustering with growth events, clustering without growth events, and nonevents. Lower and upper quartile of the boxes represent 25th and 75th percentiles, respectively. Large positive ions, i.e., mass-to-charge ratio >500 Th (which were labeled as "unidentified" in panel (a) but likely to be organics (*12*)) exhibited the highest signals for clustering with growth events, and lowest for nonevents.

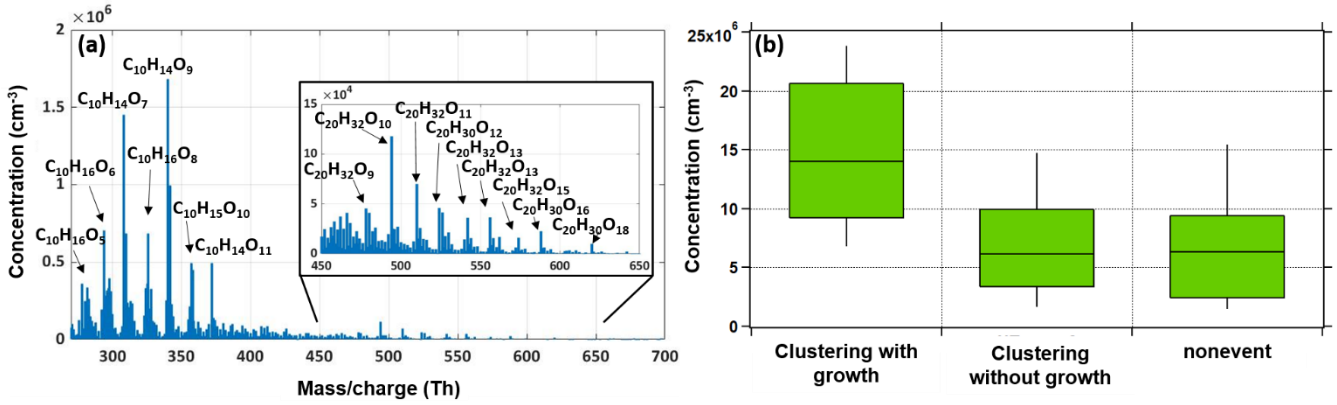

Fig. S8. Gaseous concentration of HOM molecules measured by CI-APi-ToF. **(a)** Concentration difference of different HOM molecules for clustering with growth events and clustering without growth events. **(b)** Concentration of HOM molecules most contributing to growth for clustering with growth events, clustering without growth events, and nonevents. Lower and upper quartile of the boxes represent 25th and 75th percentiles, respectively. Higher concentrations of low volatility vapors (i.e., neutral HOM) are required to facilitate the further growth of clusters.

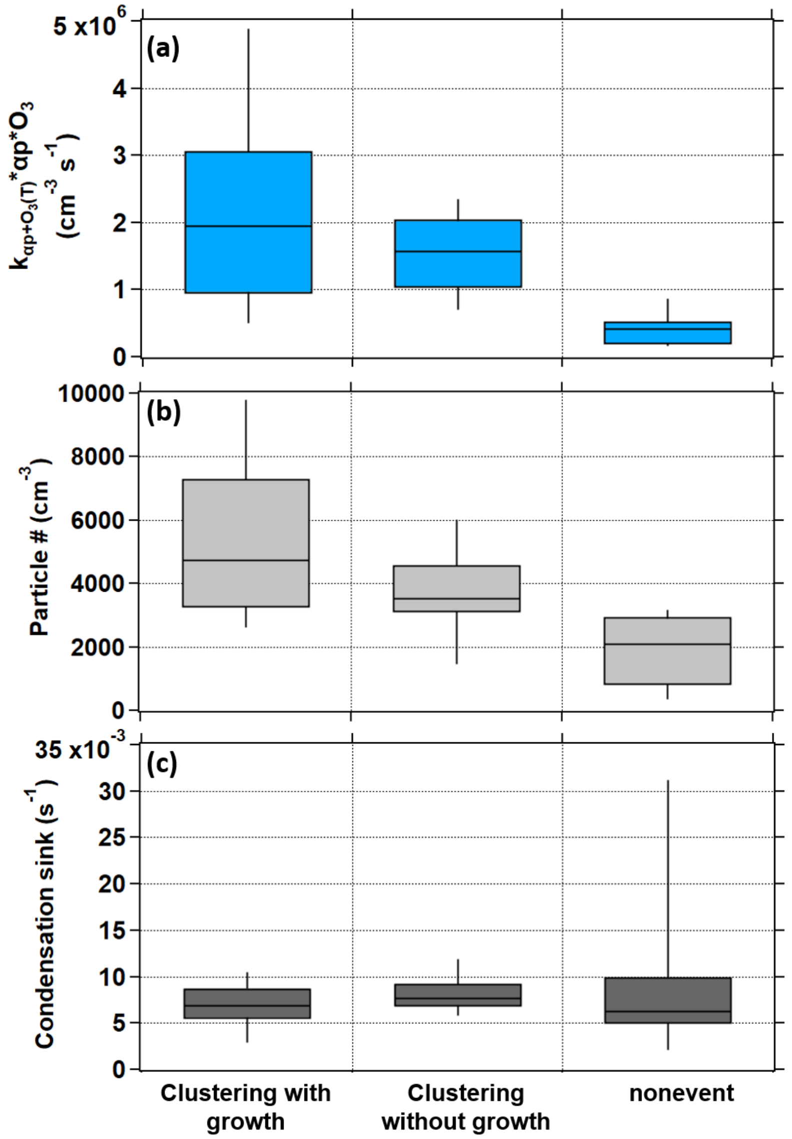

Fig. S9. Comparison of particle formation related parameters for the three types of nocturnal situations in Siikaneva. (a) α-pinene reactivity by ozonolysis indicating the product formation rate (assuming all monoterpenes measured by PTR-TOF were α-pinene, as it is the dominating monoterpene in Siikaneva (*21*)). The reaction rate coefficient of α-pinene with O_3, $k_{\alpha p+O3(T)}$, is from International Union of Pure and Applied Chemistry (IUPAC, https://iupac.aeris-data.fr/en/home/). **(b)** Total number concentration for particles with mobility diameters larger than 2.5 nm measured with the CPC3776, and **(c)** condensation sink level, which is calculated from a differential mobility particle sizer (DMPS) measurement in Hyytiälä (*15*). Lower and upper quartile of the boxes represent 25th and 75th percentiles, respectively. Highest levels of total HOM for clustering with growth events is due to the higher α-pinene reactivity with O_3 and lower condensation sink level. This led to higher (new) particle number concentrations as shown in (b).

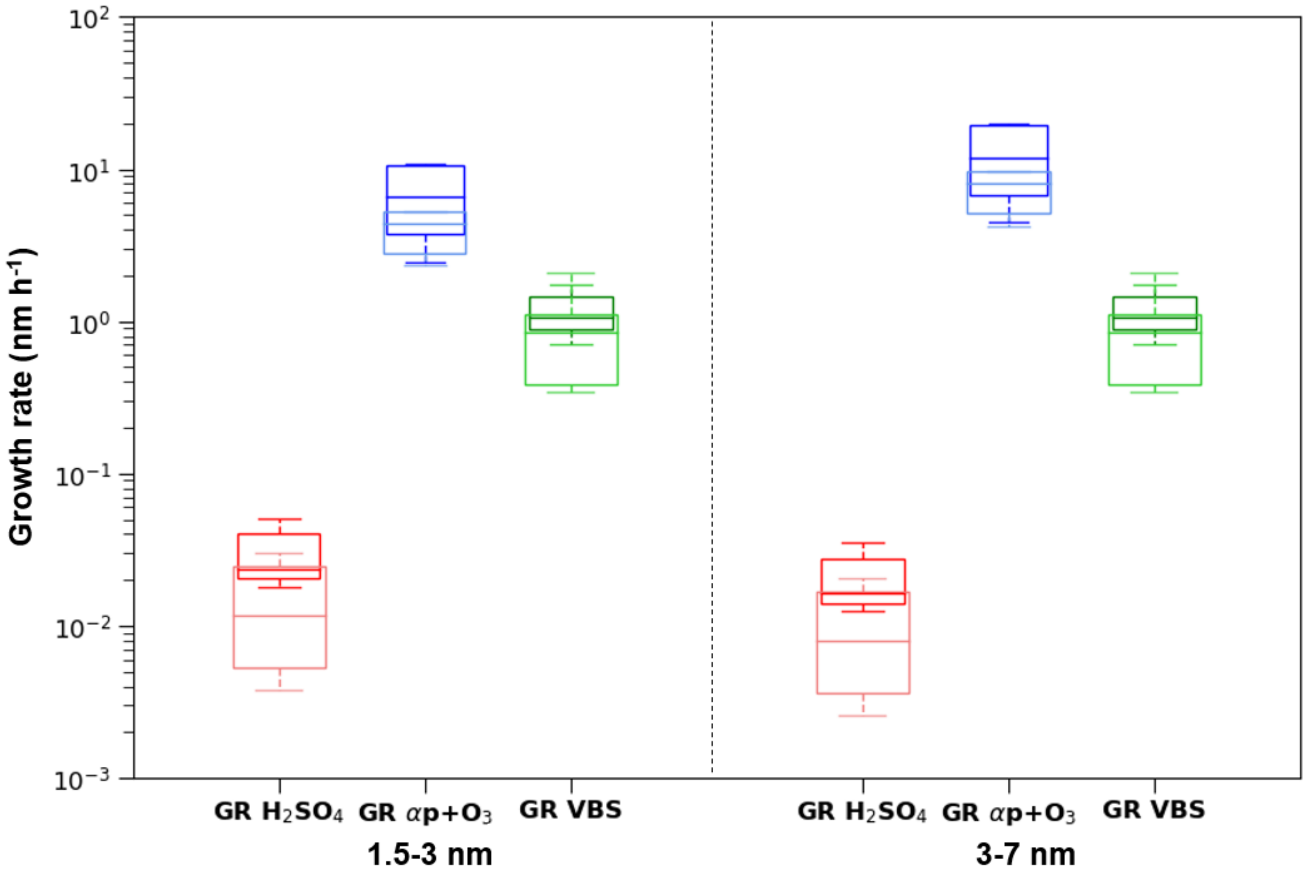

Fig. S10. Growth rates (GR) for clustering without growth events compared to those with growth events in Siikaneva. Comparison of the GR for clustering with growth events (dark color) and without growth events (light color), calculated by assuming that solely sulfuric acid contributes to the growth (*31*) (red), or by also assuming a contribution from α-pinene (αp) ozonolysis reactivity (*34*) (blue), or directly from measured HOM via VBS (volatility basis set) calculation (*33*) (green). Lower and upper quartile of the boxes represent 25th and 75th percentiles, respectively. The potential GR calculated from organic concentrations for the clustering without growth events was on average a factor of ~2 lower than those for the clustering with growth events, for both methods and size ranges.

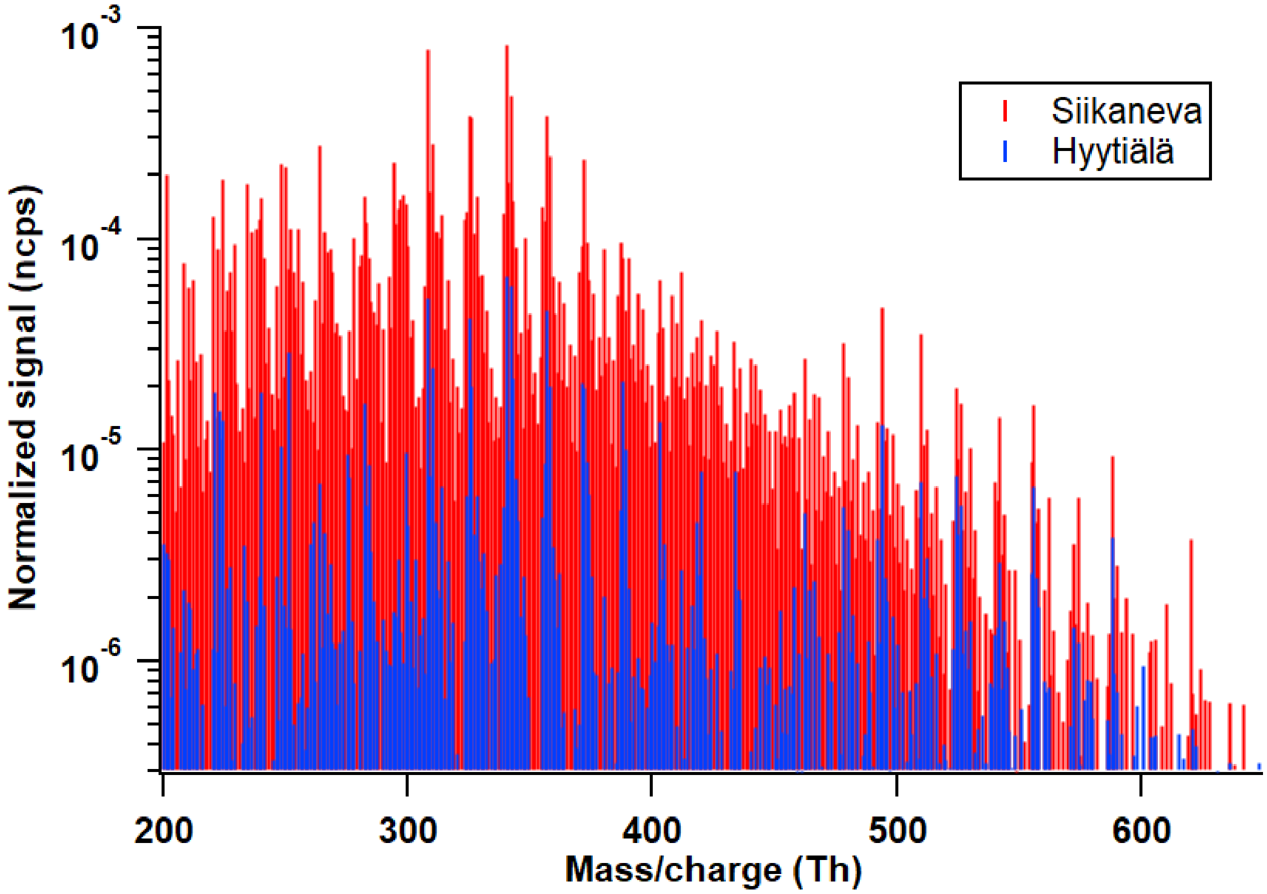

Fig. S11. Comparison of HOM spectra measured by CI-APi-ToF for a nighttime clustering with growth event in Siikaneva and a nighttime clustering without growth event in Hyytiälä. Data are from the same CI-APi-ToF. HOM spectra were very similar for the clustering events at both sites. However, higher HOM levels were observed for the clustering with growth event in Siikaneva (May 20, 2016) than those for the clustering without growth event in Hyytiälä (April 18, 2017), indicating higher concentrations of low volatility vapors (i.e., HOM for our case) are required to facilitate the further growth of clusters (see also Fig. S8). This is also similar to the comparison of HOM levels between clustering with growth events and without growth events in Siikaneva.

Table S1. Classification of events used for data analysis of charged clusters (measured with APi-TOF) and neutral clusters (measured with CI-APi-TOF).

count	Clustering with growth	Clustering without growth	nonevent
1	20160503 20:00-23:00	20160507 00:00-06:00	20160504 20:00-23:00
2	20160511 20:00-00:00	20160507 20:00-06:00	20160508 20:00-23:00
3	20160520 19:00-23:00	20160509 00:00-06:00	20160510 20:00-23:00
4	20160522 20:00-23:00	20160509 20:00-06:00	20160513 20:00-23:00
5	20160523 20:00-00:00	20160514 03:00-06:00	20160515 20:00-23:00
6	20160524 19:00-22:00	20160519 22:00-05:00	20160518 20:00-23:00
7	**20160529 20:00-22:00**	20160520 23:00-05:00	20160521 20:00-23:00
8	**20160530 20:00-23:00**	20160525 00:00-04:00	20160526 20:00-23:00
9	**20160531 20:00-00:00**	**20160527 23:00-03:00**	**20160528 20:00-23:00**
10		**20160529 22:00-06:00**	**20160607 20:00-23:00**
11		**20160602 20:00-05:00**	**20160608 20:00-23:00**
12		**20160604 22:00-23:00**	**20160609 20:00-23:00**
13		**20160606 23:00-04:00**	**20160618 20:00-23:00**
14		**20160615 21:00-22:00**	**20160619 20:00-23:00**

Events in bold are used for positive mode API data analysis and black are used for negative mode API data analysis due to the different period of modes used.
